# Preclinical evaluation of KIT/PDGFRA and mTOR inhibitors in gastrointestinal stromal tumors using small animal FDG PET

**DOI:** 10.1186/1756-9966-29-173

**Published:** 2010-12-30

**Authors:** Maria Abbondanza Pantaleo, Giordano Nicoletti, Cristina Nanni, Chiara Gnocchi, Lorena Landuzzi, Carmelo Quarta, Stefano Boschi, Margherita Nannini, Monica Di Battista, Paolo Castellucci, Stefano Fanti, Pier Luigi Lollini, Elena Bellan, Mauro Castelli, Domenico Rubello, Guido Biasco

**Affiliations:** 1 Department of Hematology and Oncology Sciences "L.A.Seragnoli", Sant' Orsola-Malpighi Hospital, University of Bologna, Bologna, Italy; 2 Laboratory of Experimental Oncology, Istituto Ortopedico Rizzoli, Bologna, Italy; 3 Nuclear Medicine Service, Sant' Orsola-Malpighi Hospital, University of Bologna, Bologna, Italy; 4 Novartis Oncology, Origgio, Italy; 5 PET Radiopharmacy-Nuclear Medicine Service, Sant' Orsola-Malpighi Hospital, University of Bologna, Italy; 6 Interdepartmental Centre of Cancer Research "G. Prodi", University of Bologna, Italy; 7 Service of Medical Physics, Santa Maria della Misericordia Hospital, Rovigo, Italy; 8 Department of Experimental Oncology, Regina Elena National Cancer Institute, Roma, Italy; 9 Department of Nuclear Medicine, Santa Maria della Misericordia Hospital, Rovigo, Italy

## Abstract

**Background:**

Primary and secondary drug resistance to imatinib and sunitinib in patients with gastrointestinal stromal tumors (GISTs) has led to a pressing need for new therapeutic strategies such as drug combinations. Most GISTs are caused by mutations in the KIT receptor, leading to upregulated KIT tyrosine kinase activity. Imatinib and nilotinib directly inhibit the kinase activity of KIT, while RAD001 (everolimus) inhibits mTOR. We report a preclinical study on drug combinations in a xenograft model of GIST in which effects on tumor dimensions and metabolic activity were assessed by small animal PET imaging.

**Methods:**

Rag2-/-; γcommon -/- male mice were injected s.c. into the right leg with GIST 882. The animals were randomized into 6 groups of 6 animals each for different treatment regimens: No therapy (control), imatinib (150 mg/kg b.i.d.) by oral gavage for 6 days, then once/day for another 7 days, everolimus (10 mg/kg/d.) by oral gavage, everolimus (10 mg/kg/d.) + imatinib (150 mg/kg b.i.d.) by oral gavage for 6 days, then once/day for another 7 days, nilotinib (75 mg/kg/d.) by oral gavage, nilotinib (75 mg/kg/d.) + imatinib (150 mg/kg b.i.d) by oral gavage for 6 days, then once/day for another 7 days. Tumor growth control was evaluated by measuring tumor volume (cm^3^). Small animal PET (GE Explore tomography) was used to evaluate tumor metabolism and performed in one animal per group at base-line then after 4 and 13 days of treatment.

**Results:**

After a median latency time of 31 days, tumors grew in all animals (volume 0,06-0,15 cm^3^) and the treatments began at day 38 after cell injection. Tumor volume control (cm3) after 13 days of treatment was > 0.5 for imatinib alone and nilotinib alone, and < 0.5 for the 2 combinations of drugs and for everolimus alone. The baseline FDG uptake was positive in all animals. FDG/SUV/TBR was strongly reduced over time by everolimus both as a single agent and in combination with imatinib respectively: 3.1 vs. 2.3 vs. 1.9 and 2.5 vs 2.3 vs 0.

**Conclusions:**

As single agents, all drugs showed an anti-tumor effect in GIST xenografts but everolimus was superior. The everolimus plus imatinib combination appeared to be the most active regimen both in terms of inhibiting tumor growth and tumor metabolism. The integration of everolimus in GIST treatment merits further investigation.

## Introduction

Gastrointestinal Stromal Tumors (GISTs) are a rare malignancy originating from Cajal's cells of the gastrointestinal tract. Most GISTs are caused by mutations in the KIT and PDGFRA receptors, leading to upregulated tyrosine kinase activity [1,2]. Tyrosine kinase inhibitors (TKIs), imatinib and sunitinib, are the standard treatment for patients with advanced or unresectable GIST [3,4]. However, the occurrence of primary and secondary drug resistance to TKIs has led to a pressing need to develop new drugs or new strategies such as drug combinations [5-7]. Nilotinib is a second-generation multitarget TKI that directly inhibits the kinase activity of KIT and PDGFRA receptors and also BCR-ABL, PDGFRA and KIT [8]. Nilotinib has been shown to be active in a small series of patients pre-treated with imatinib and sunitinib [9,10]. RAD001 (everolimus) inhibits the mammalian target of rapamycin (mTOR) which is involved in various intracellular signaling pathways and represents a therapeutic target for treatments of solid tumors [11,12]. mTOR may be activated as an alternate oncogenic signaling mechanism in TKI resistance and mTOR inhibitors have yielded interesting results in GIST even if they emerged from small series of patients [13-18]. The rationale of the TKIs and RAD001 combination derives from an *in vitro *demonstration on resistant GIST cell lines where everolimus associated with imatinib had a synergic antitumor effect. The combination of TKIs and mTOR inhibitors may be promising for a more complete inhibition of the KIT/PDGRA signaling pathway and a better tumor response.

As is well known from the clinical setting, the tumor response still cannot be evaluated using the traditional RECIST (Response Evaluation Criteria in Solid Tumors) alone because mostly TKIs do not lead to lesion shrinkage [19-21]. Therefore, the CHOI criteria have been studied using both tumor size and density variations to evaluate GIST lesions treated with imatinib [22]. As a result, the preclinical development of new drugs or a combination of drugs and molecular targets should be planned with a modern approach based on tumor dimensions and metabolic activity evaluation [23,24]. We recently developed a xenograft model of GIST measuring tumor metabolism using small animal PET imaging [23].

The aim of this work is to report a preclinical study on the antitumor activity of drug combinations, TKIs and m-TOR inhibitors, in a xenograft model of GIST in which the drug effects were assessed by small animal PET imaging evaluating both tumor growth control and tumor glucose metabolism.

## Materials and methods

### Experimental model

Tumor xenografts were developed with the GIST882 cell line provided by Dr. Jonathan A. Fletcher, Harvard Medical School, Boston, Massachusetts, USA.

All data on the GIST882 cell line, cytofluorometric studies and KIT and PDGFRA mutational analysis of GIST882 cells showing a mutation on KIT receptor exon 13 (homozygous mutation - K642E) were reported in our previous article [23]. Rag2^-/-^;γc^-/- ^breeders were kindly given by Drs. T. Nomura and M. Ito of the Central Institute for Experimental Animals [25]; mice were then bred in our animal facilities under sterile conditions. The experiment was authorized by the institutional review board of the University of Bologna and done according to Italian and European guidelines.

Tumor xenografts were induced into Rag2^-/-^;γc^-/- ^male mice by subcutaneous (s.c.) injection of 10^7 ^viable GIST882 cells in 0.2 ml phosphate-buffered saline (PBS) into the right leg. Tumor incidence and growth were evaluated three times a week. Neoplastic masses were measured with calipers; tumor volume was calculated as π. [√(*a. b)*]^3^/6, where *a *= maximal tumor diameter and *b *= tumor diameter perpendicular to *a*.

Two months after cell injection mice were sacrificed by CO_2 _inhalation and necropsied.

### Treatments protocols

Animals were randomized into 6 groups of 6 animals each one for different treatment regimens which were given for 13 days:

* No therapy (control)

* Imatinib (150 mg/kg b.i.d.) by oral gavage for 6 days, then once/day for another 7 days

* Everolimus (10 mg/kg/d.) by oral gavage

* Everolimus (10 mg/kg/d.) + imatinib (150 mg/kg b.i.d.) by oral gavage for 6 days, then once/day for another 7 days

* Nilotinib (75 mg/kg/d.) by oral gavage

* Nilotinib (75 mg/kg/d.) + imatinib (150 mg/kg b.i.d) by oral gavage for 6 days, then once/day for another 7 days

### Imaging studies

Imaging studies were performed using a small animal PET tomograph (GE, eXplore Vista DR) using fluoro-deoxyglucose (FDG) for glucose metabolism. Animals had PET scans after gas anaesthesia (sevofluorane 3-5% and oxygen 1 l/min). FDG was injected into a tail vein. FDG uptake was evaluated by standard uptake value (SUV)/tumor background ratio (TBR). PET scans were performed in one animal per group at base-line, and after 4 and 13 days of treatment.

## Results

**Figure 1:**
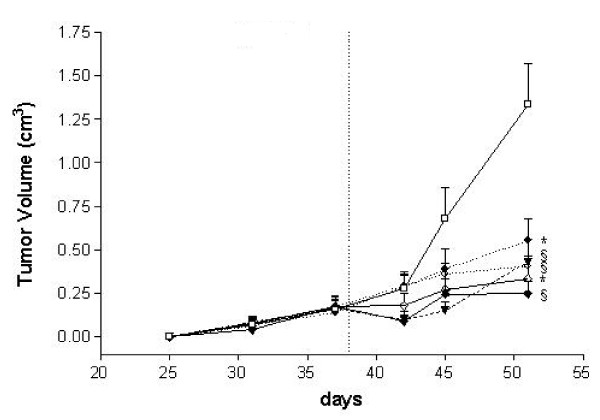
**Inhibition of tumor growth in Rag2-/-; γcommon -/- male mice injected s.c. with GIST 882 by treatment p.o. with untreated (-□-), imatinib (-◊-), everolimus (⋯○⋯), imatinib+everolimus (-♦-), nilotinib (⋯●⋯), nilotinib+imatinib (--▼-)**. The dotted line marks the beginning of therapy. The tumor volumes are expressed as mean ± E.S in cm^3^. ^§^p > 0.01, *p < 0.05, Student's t test compared with untreated group.

After subcutaneous injection, tumors grew very slowly and sometimes indolently (median latency time: 31 days) in all animals (volume 0,06-0,15 cm^3^). The treatments began at day 38 after cell injection when all animals were tumor bearing. The mice were randomly distributed in the 6 experimental groups to have the same mean tumor volume in all experimental groups at the start of treatment (Figure 1).

Before starting treatments, the in vivo tumor mass was evaluated using small animal PET tomography in one animal per group (37 days after cell injection). The base-line FDG uptake was positive in all animals evaluated with a mean SUV/TBR of 2.78 (range 3.12-2.23).

In the 6 groups, only three animals out of the 36 died during the protocol, two in the imatinib group, and one in everolimus + imatinib group. The efficacy of the treatments was evaluated at first as effect on tumor growth (dimensions measured by calipers). All treatments were statistically different (at least p > 0.05) when compared with the untreated group.

**Figure 2:**
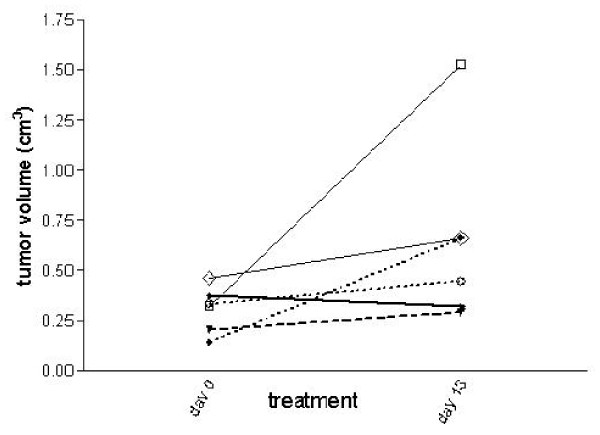
**Tumor volume of the same animal per group also examined by PET scan**. The points indicate tumor volume, measured with calipers, expressed in cm^3 ^at day 0 and at day 13 of treatment. In imatinib group the tumor volumes refer to two different animals. Rag2-/-; γcommon -/- male mice injected s.c. with GIST 882 were treated p.o. with untreated (-□-), imatinib (-◊-), everolimus (⋯○⋯), imatinib+everolimus (-♦-), nilotinib (⋯●⋯), nilotinib+imatinib (--▼-).

After 4 and 13 days of treatment, one representative animal for each group was evaluated either with calipers to measure tumor size (tumor volume expressed in cm^3 ^at days 0 and 13 of treatments is shown in Figure 2) and with PET tomography. At day 13, the mean tumor volume of all animals per group was > 0.5 cm^3 ^for imatinib alone and nilotinib alone, and < 0.5 cm^3 ^for the 2 combinations and for everolimus alone.

**Figure 3:**
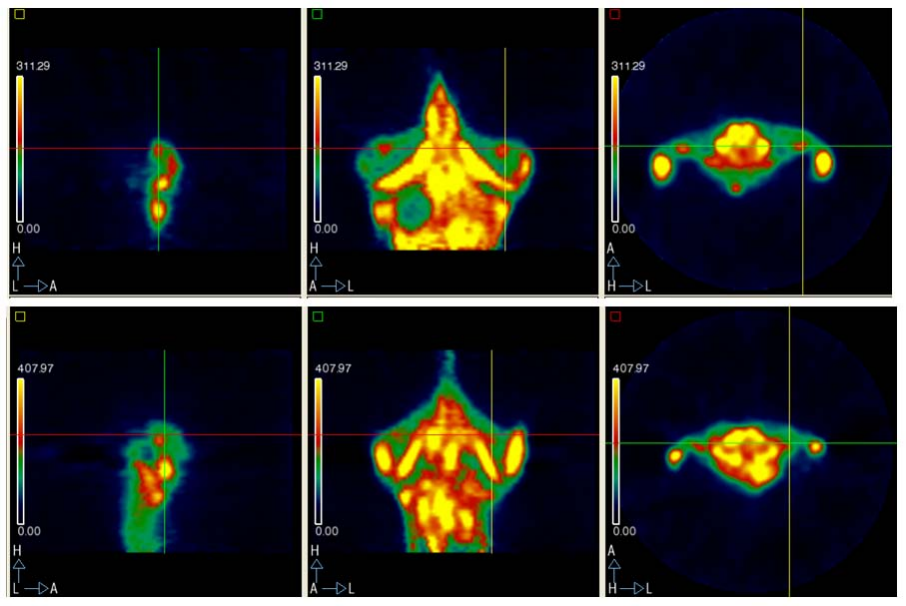
**Small animal PET images for everolimus as a single agent: pre-treatment lateral (A), coronal (B) and axial (C) SUV TBR 3.12; post-treatment lateral (D), coronal (E) and axial (F) SUV TBR 1.98**.

**Figure 4:**
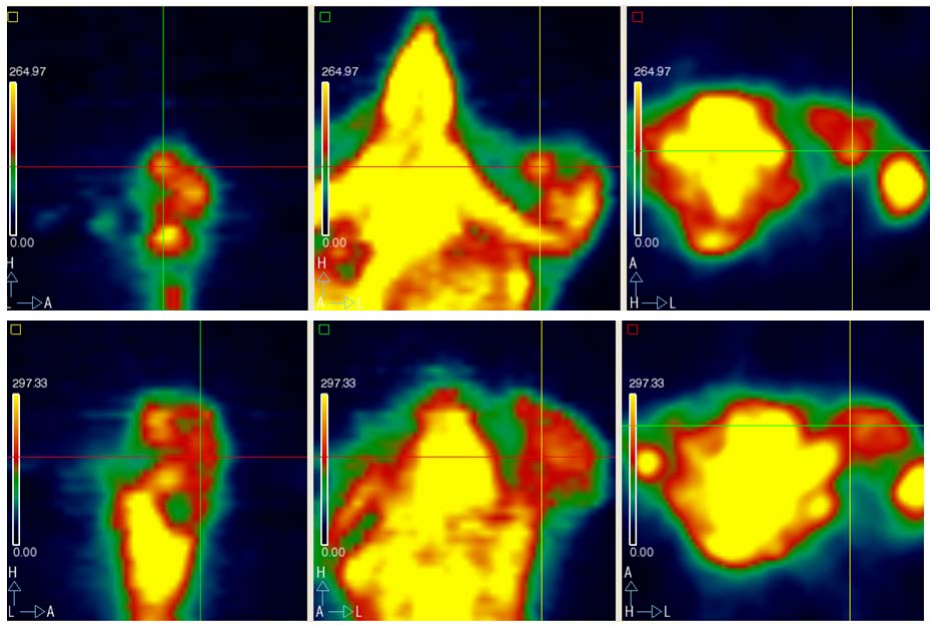
**Small animal PET images for everolimus combined with imatinib: pre-treatment lateral (A), coronal (B) and axial (C) SUV TBR 2.59; post-treatment lateral (D), coronal (E) and axial (F) SUV no uptake**.

SUV/TBR at base line and after 4 and 13 days of treatments was:

* Control: 3.08 base line; 2.19 (large necrosis) after 4 days; 1.19 (large necrosis) after 13 days

* Imatinib: 2.91; 2; 2.53

* Everolimus: 3.12; 2.3; 1.98 * Everolimus and imatinib: 2.59; 2.23; 0 (Figure 3)

* Nilotinib: 2.23; 1.42; 1.7 (Figure 4)

* Nilotinib + imatinib: 2.76; 3.28; 2.83;

The mouse in the imatinib group that had the first baseline and the second PET scan after treatment died during the protocol and the third PET scan was performed in a second animal; this new animal was comparable to the first one for tumor growth. Everolimus strongly reduced FDG uptake both alone and in combination with imatinib.

## Discussion

Despite the dramatic results in disease control by TKIs in GIST, patients may develop primary and secondary drug resistance and this has led to a pressing need to develop new drugs or new strategies such as drug combinations.

We have developed a xenograft model of GIST suitable for the preclinical study of new treatments evaluating both tumor size and function. This experiment used the model to study the antitumor activity of drug combinations, TKIs and m-TOR inhibitors [23]. We studied the activity of everolimus as a new single agent and two combinations of agents, imatinib associated with nilotinib and imatinib associated with everolimus. Imatinib and nilotinib as single agents were also evaluated for comparison and a non-treated group of animals served as a general control. As single agents all 3 drugs controlled tumor growth. Everolimus alone was superior to nilotinib and imatinib (tumor volume (cm^3^) after 13 days of treatment: 0.4 vs 0.6 vs 0.6 respectively). Both combined regimens were more effective than single drugs (both 0.3 cm^3 ^vs > 0.4 cm^3^). Considering tumor glucose metabolism, the control group showed a reduction of FDG SUV value due to the progressive development of necrosis due to a massive increase in tumor size. The imatinib group cannot be considered because the mouse subjected to the first 2 PET scans died before the third scan. All the other therapeutic regimens showed a reduction of FDG SUV value after treatment administration, except the nilotinib and imatinib combination where the FDG SUV value remained stable. Attention should be paid to the everolimus and imatinib combination where FDG uptake was progressively reduced until there was no uptake after 13 days (SUV 2.59; 2.23; 0) (Figure 3).

Everolimus showed the most interesting results in our experiment as it had an antitumor effect both as a single agent and in combination with imatinib, considering both tumor volume control and inhibition of glucose metabolism. FDG was strongly reduced by everolimus alone and combined with imatinib. Everolimus inhibits mTOR which is a KIT/PDGFRA downstream pathway-dependent target and seems to be a promising agent in GIST. Other preclinical data on everolimus in a GIST cell line were reported by Chang et al with the evaluation of treatment response in the GIST 882 cell line by the reduction of phospho-AKT and phospho-S6 after imatinib and everolimus [26]. In a clinical setting, everolimus associated with imatinib was used in small series of patients [13,14,17,18]. A phase I-II trial of everolimus (RAD001) at a dose of 2.5 mg in combination with imatinib 600 mg daily achieved a progression-free survival of at least 4 months in imatinib-resistant GIST patients after first- and second line-treatment failure [14]. Sirolimus, another mTOR inhibitor, in association with TKIs (PKC412 or imatinib) showed an antitumor activity in three GIST patients harbouring exon 18 PDGFRA-D842V mutation, that is well known to confer resistance to imatinib *in vitro *and *in vivo *[15,16]. This combination is interesting because it simultaneously inhibits two different molecules of the same signaling pathway (KIT-PDGFRA/PI3-K/AKT/mTOR) that impacts on cancer cell growth, survival, motility and metabolism [27].

Nilotinib is a second-generation multi-TKI inhibitor that showed 7 to 10-fold higher intracellular concentrations than imatinib *in vitro *[28]. This feature may be important to overcome the reduced affinity of the binding between imatinib and TK due to the acquisition of new mutations and to avoid the problem of an up-regulation of efflux transporters. Nilotinib achieved a median progression-free survival of 12 weeks and a median overall survival of 34 weeks in a small series of patients pre-treated with imatinib and sunitinib [9]. An *in vitro *and *in vivo *study on V561D-PDGFRA and D842V-PDGFRA mutants demonstrated that the combinations of nilotinib, imatinib and PKC412 could have a cooperative anti-proliferative activity due to their synergic effects on multiple targets [29]. A clinical study reported that nilotinib alone or in combination with imatinib was well tolerated overall and showed clinical activity in 53 imatinib-resistant GIST patients in terms of median progression-free survival (203 days vs 168 days) and median duration of disease control (259 vs 158 days) [30]. A large phase III trial on nilotinib as monotherapy in pre-treated GIST patients has been completed and, moreover, a large phase III trial comparing imatinib versus nilotinib in untreated metastatic patients is still ongoing [10,31]. In our experiment, nilotinib as a single agent showed the same results as imatinib in tumor volume control, but it also led to a good reduction of FDG uptake reduction over time. However, the combination with imatinib is superior to the single agent alone. Moreover, nilotinib combined with imatinib showed the same results as the regimen imatinib and everolimus, but tumor metabolism after treatment was stable and hence the FDG uptake reduction was less evident than with imatinib and everolimus. In general our report confirms the effect of nilotinib in GIST treatment, and no further preclinical studies of nilotinib as a single agent or combined with imatinib are necessary. We still have to wait for more data from clinical trials in order to define the activity and safety profile of this drug and its role in the treatment of GIST patients. When these data are available, an interesting clinical evaluation may focus on the combination of nilotinib with mTOR inhibitors.

To date, no one combination of agents has yet been approved as standard GIST treatment in clinical practice. However, there is a growing interest in combined therapies for various reasons [27], the commonest being the occurrence of primary and secondary resistance related to KIT and PDGFRA kinase genotype status [5,6]. Specific point mutations are associated with a different sensitivity to imatinib. Wild-type KIT/PDGFRA GISTs are also generally more resistant to imatinib. KIT or PDGFRA receptor abnormalities including KIT gene amplification, loss of KIT expression, and acquired mutations interfering with imatinib binding may also occur. Many cases of GIST show a clonal progression of disease with different nodules harbouring different KIT and PDGFRA mutations that confer an inter- and intra-lesional heterogeneity of drug resistance [32]. Moreover, new KIT/PGDFRA-dependent molecular targets, such as PI3K, AKT, mTOR, BRAF. and KIT-independent pathways such as IGF-1R, VEGF have been discovered in GIST and should be integrated in the therapeutic approach to overcome drug resistance [27]. Lastly, histological changes, chromosomal alterations or a decrease of imatinib bioavailability may affect TKs responsiveness.

Apart from the combinations of different TKIs and mTOR inhibitors discussed above, other potential combinations in GIST have been reported. The addition of perifosine, an AKT inhibitor, to imatinib showed a minimal activity in 40 imatinib-resistant GIST patients, but 4/5 (80%) patients with WT GIST experienced 1 partial response and 3 had stable disease according to Choi's criteria [33]. A phase III randomized trial of imatinib, with or without bevacizumab (SO502 trial) in untreated patients with metastatic or unresectable GIST is now ongoing. As future perspectives, IGF-1R inhibitors should be combined with TKIs because IGF1r was recently found over-expressed in GISTs, especially in children and WT young adults GISTs patients [34-38].

Potential therapeutic combinations are growing, but more preclinical studies of these strategies using adequate models are needed. Cell lines well characterized for the molecular and genomic background, and sophisticated xenograft animals of GIST are required to study the mechanism of drug activity or drug-mediated up or down-regulated molecular profiles and the acquisition of secondary biological aberrations. Recently, knock-in murine animals were bred by introducing a germ-line gain-of-function mutation of the KIT receptor into the mouse genome [39-43]. The future correlation between small animal imaging features and molecular analyses may held to clarify the antitumor effect of new therapeutic strategies before clinical implementation.

In conclusion, we report the in vivo evaluation of antitumor activity of single agents and combined treatments in GIST. All drugs were active as single agents, but everolimus was superior. The two drug combinations showed a better control of tumor growth than single agents. The everolimus plus imatinib combination was the most active regimen both in terms of inhibiting tumor growth and FDG reduction, and represents the most exciting therapeutic perspective for treatments in GISTs.

## Competing interests

The authors declare that they have no competing interests.

## Authors' contributions

MAP, GN, CG, LL, MN, MDB, PLL corrected the data and performed the laboratory tests; moreover contribute to prepare the draft of the manuscript; CN, CQ, PC, EB performed PET examinations, moreover contribute to prepare the draft of the manuscript; SF, GB, MC, DR conceived the study, participated in its design and coordination. All authors read and approved the final manuscript.
